# A Delayed Diagnosis: Conflicting Investigation Results Should Be Challenged

**DOI:** 10.1155/2011/809567

**Published:** 2011-09-22

**Authors:** D. Mital, V. Desai

**Affiliations:** Department of Sexual Health and HIV Medicine, Milton Keynes NHS Foundation Trust, Standing Way, Milton Keynes MK6 5LD, UK

## Abstract

We present a case where a diagnosis of advanced HIV led to many other diagnoses but more importantly life-threatening multivariant Castleman's disease which is rare and was successfully treated. This case highlights the importance of questioning and challenging investigations when it does not fit with the clinical picture. Multidisciplinary teams are crucial and always seek expert advice if unsure. The much-quoted adage of “many symptoms and signs will fit one diagnosis” also bodes well in this case.

## 1. Lesson

A 43-year-old Caucasian German Salesman was referred to our outpatient clinic from the Haematologists in July 2009. He was under their care for anaemia and had lost 10 kg of weight in 6 months. He also had increasing shortness of breath on exertion with a cough productive of yellow phlegm, increasing lethargy, dizziness on standing, and numbness in both hands. His wife had HIV infection for 6 years but had only recently disclosed her diagnosis to him. They have been together for 11 years. He had a minimal past medical history with previous knee cartilage repair only and no family history or children. He has travelled to Mauritius, Spain, and the Dominican Republic in the past and denies smoking, drinking very much alcohol, or indulging in recreational drugs. He denied other HIV risk factors including male partners, blood transfusions, or female partners from risk countries. 

Examination revealed a thin gentleman with multiple supraclavicular, axillary, and inguinal lymph nodes which were soft, nonmotile, and up to 2 cm in size. He had hepatosplenomegaly with a 1 cm liver edge and a 4 cm spleen palpated. His chest was clear and the neurological examination normal. Investigations showed a haemaglobin of 8.6 g/dl, platelets 186, MCV 76.4 IU/l and a neutropenic picture (1.3 IU/l). His PT was prolonged at 15.7 seconds, but his LFTs were normal except for a low albumin of 27 IU/l. He had raised globulin (74 IU/l) and total protein (100 IU/l) levels with an ESR of 122 IU/l and a CRP of 22 IU/l. The haemoglobin electrophoresis showed raised IgG lambda paraprotein and kappa light chains. He was counselled and consented for an HIV-1 test which was positive. His vitamin B12 levels were low at 112 IU/l despite a microcytic picture. 

His CD4 count was 140 cells/*μ*l and the HIV-1 viral load was 710,000 copies/ml which suggested advanced HIV-1 infection and profound immunosuppression. A sputum culture grew yeasts only with no AAFB. The chest X-ray showed mediastinal and hilar lymphadenopathy with a subsequent CT chest/abdomen/pelvis revealing multiple, enlarged lymph nodes in the axilla, subcarinal, para-aortic, retroperitoneal, and mesenteric areas with a 14.5 cm spleen enlarged to the right iliac crest. He was referred to the ENT team for a cervical lymph node biopsy.

**Figure 1:**
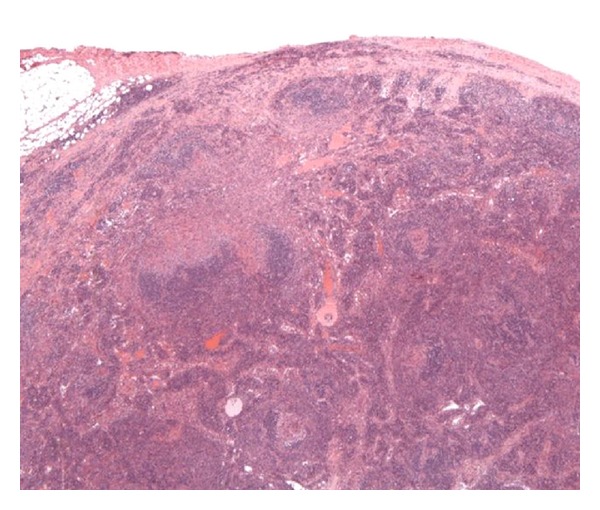
Low power view of Lymph Node with plasma cell variant castleman's and showing features of Kaposi sarcoma at the top left quadrant.

**Figure 2:**
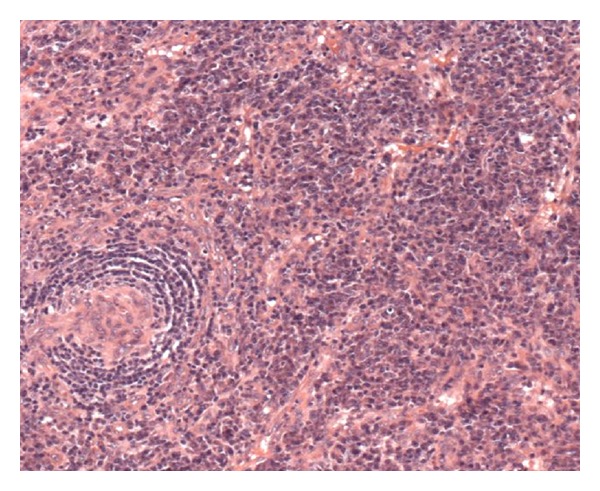
High power view of the node with plasma cell variant of castlemans.

He was commenced on Truvada and Efavirenz as standard triple antiretroviral therapy for HIV-1 infection after a full discussion on the importance of adherence, possible toxicities', and side effects. Daily 480 mg cotrimoxazole orally was started as part of *Pneumocystis Jiroveci Pneumoni *(PCP) prophylaxis, fluconazole for possible disseminated fungal infection and standard intramuscular hydroxycobalamin given for the vitamin B12 deficiency. The lymph node biopsy (see Figures 1 and 2) report returned as “*Atrophic follicles, germinal centre involution, lymphocytic depletion in paracortex:* (i)* +ve staining for kappa + lambda, *(ii)* appearances compatible with subacute/chronic stage of HIV lymphadenitis, and *(iii) *no evidence of lymphoma or carcinoma seen.*” 

At a 2-week review, he showed a 2 log drop in his HIV-1 viral load but complained of an itchy rash, and thus, his cotrimoxazole was switched to dapsone. Further results showed a positive latent syphilis result which was treated with 3 doses of intramuscular benzathine over 3 weeks. At a 4-week review, however, he complained of flu-like symptoms, fevers, dizzy spells, and periodic faints. His hb dropped further to 7.3 g/dl with severe renal impairment having a urea of 21.4 IU/l, creatinine 221 IU/l, and a corrected calcium of 1.94 IU/l. A Fanconi's syndrome secondary to the Tenofovir component of his antiretroviral therapy was the likely diagnosis and was switched to Abacavir instead. A penicillin allergy was also questioned, so the 3rd dose of benzathine was not given, and he was given oral 200 mg Docycycline once daily for 28 days instead for the latent syphilis. Immune reconstitution inflammatory syndrome (IRIS) was also a possibility, but his repeat CD4 count was just 100 cells/*μ*l. He was admitted to the medical ward 3 days later as a result of worsening shortness of breath and lethargy, and although he had an improved renal function (urea 15.9 IU/l and creatinine 171 IU/l), he had pancytopenia with his hb 6.6, wcc 0.6 IU/l, platelets 97 IU/l, and 0.0 neutrophils! He was given 4 units of blood and standard neutropenic antibiotic cover. He developed violaceous lesions over his chest wall and left thigh which looked like Kaposi's sarcoma (KS), and as his hb was still low at 7.0 IU/l with normal B12/folate/ferritin, KS was thought to be in his chest and GI systems. 

A repeat CT chest/abdomen/pelvis with contrast was done which showed 

*“There is extensive lymphadenopathy above and below the diaphragm with enlarged axillary, bilateral hilar, subcarinal, paraaortic, small bowel, mesenteric and inguinal nodes, up to 2.5* *cm in the para-aortic region. There is peribronchial thickening in the right middle lobe with small bilateral pleural effusions in conjunction with right hilar lymphadenopathy? pulmonary Kaposi's sarcoma + increased splenomegaly + widespread lymphadenopathy.”*

He was transferred to the John Radcliffe Hospital, Oxford, for further management, as it was felt that complex care with possible chemotherapy and radiotherapy could be given at a tertiary centre. 

**Figure 3:**
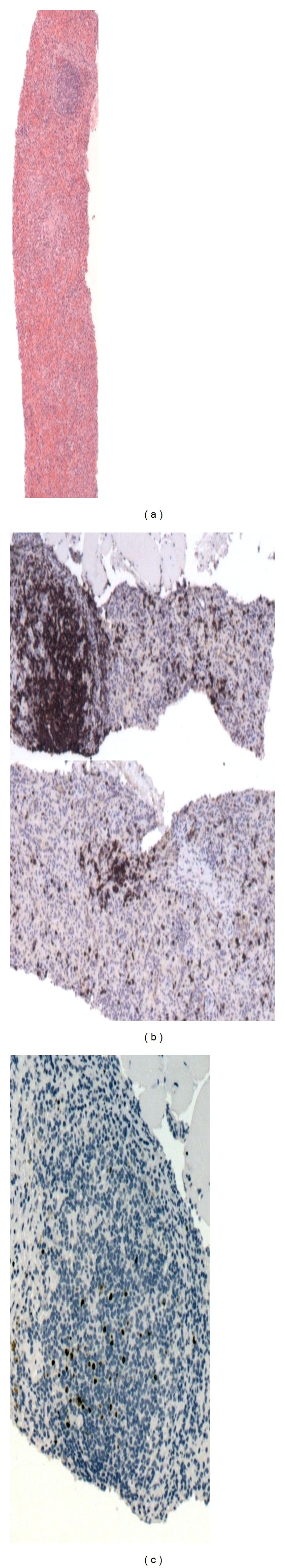
Composite picture of splenic biopsy showing H & E picture on (a), CD79a in the top and bottom (b) and HHV-8 staining cells on (c).

**Figure 5:**
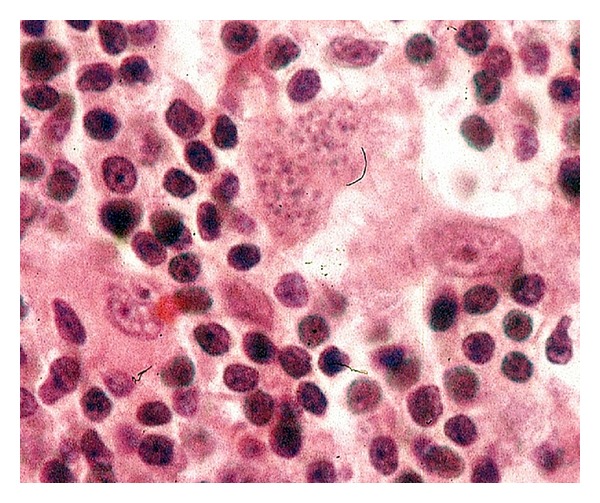
High power view of Leishmania organisms.

A splenic biopsy (Figure 3) result showed widespread Kaposi's sarcoma and plasma cell variant multifocal Castleman's disease. A bone marrow trephine biopsy also showed Leishmaniasis (Figure 5) which was treated with ambisome. The initial cervical lymphnode biopsy was relooked at, and the report suggested *“Lymph node shows changes associated with plasma cell variant of Castleman's disease. Numerous Human Herpes Virus 8 (HHV8) lymphoid cells *(Figure 5)* and vascular proliferation which are also strongly HHV8+—typically seen with cutaneous KS.”*

An oesophagoduodenoscopy and colonoscopy confirmed GI KS, but no haematemesis or malaena seen. His HHV8 viral load was high at 4,800 copies/ml. He responded very well to chemotherapy with 6 cycles of liposomal Doxorubicin 40 mg/m^2^ + 4 weekly IV Rituximab (4 courses). Apart from mild shingles which responded to high-dose acyclovir, he showed a good splenic and CT response to chemotherapy with the syphilis and leishmaniasis being successfully eradicated. His CD4 count is currently robust enough to stop dapsone therapy.

## 2. Discussion

Castleman's disease is a rare, benign disorder comprising of a nonclonal disease of the lymph nodes and angiofollicular hyperplasia but is more common in HIV/AIDS. There is usually widespread lymphadenopathy with hepatosplenomegaly, fatigue, night sweats, fever, wt-loss, anorexia, peripheral oedema, anaemia, low albumin, and peripheral neuropathy. It is associated with autoimmune hemolytic anemia, multiple myeloma, amyloidosis, and Pemphigus which can lead to non-Hodgkin's lymphoma [1]. Treatment is guided by histology type and involves combination chemotherapy and antiretroviral therapy [2]. As there is often a raised interleukin-6, novel therapies such as monoclonal anti-IL-6 have been used [3]. HHV8-driven Castleman's disease can be associated with KS [4, 5].

Thus, multiple diagnoses were seen in this patient which is not atypical of someone living with HIV/AIDS. Multidisciplinary teams are crucial in most chronic conditions, and a low threshold for seeking expert advice and facilities should be maintained. The multiple symptoms and signs “fitted” most in the unifying diagnosis of multivariant Castleman's disease. 

## Figures and Tables

**Figure 4:**
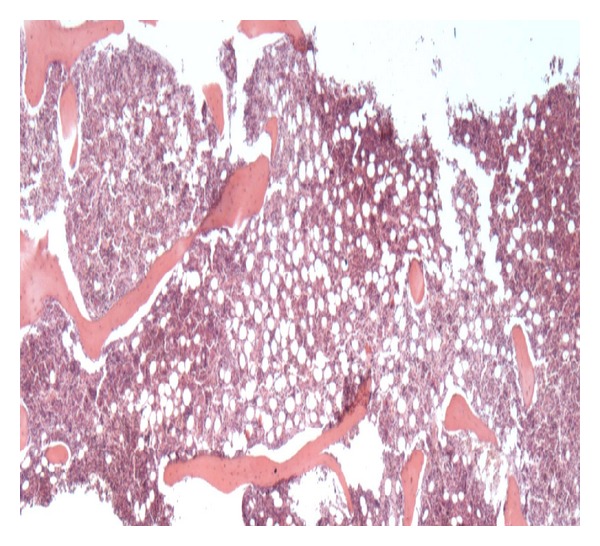
Bone marrow trephine biopsy with cellular marrow with leishmaniasis.

**Figure 6:**
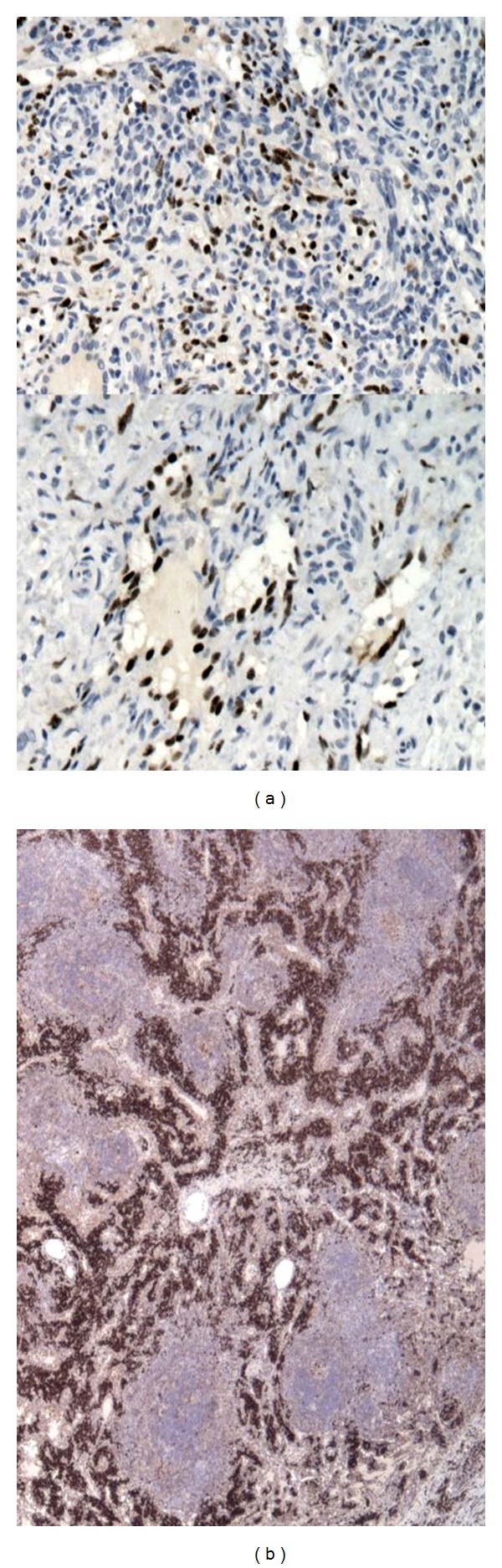
Lymph node with plasma cell marker VS 38 on (b) and HHV8 staining lymphoid cells on (a).
